# Preparation and Investigation of High Surface Area Aerogels from Crosslinked Polypropylenes

**DOI:** 10.3390/polym16101382

**Published:** 2024-05-12

**Authors:** Radek Coufal, Mateusz Fijalkowski, Kinga Adach, Huaitian Bu, Christian W. Karl, Eliška Mikysková, Stanislav Petrík

**Affiliations:** 1 Department of Science and Research, Faculty of Health Studies, Technical University of Liberec, 461 17 Liberec, Czech Republic; 2 Department of Advanced Materials, Institute for Nanomaterials, Advanced Technologies and Innovation (CXI), Technical University of Liberec, 461 17 Liberec, Czech Republic; mateusz.fijalkowski@tul.cz (M.F.); kinga.adach@tul.cz (K.A.); stanislav.petrik@tul.cz (S.P.); 3 Department of Materials and Nanotechnology, SINTEF Industry, Forskningsveien 1, 0373 Oslo, Norway; huaitian.bu@sintef.no (H.B.); christian.karl@sintef.no (C.W.K.); 4 Center for Innovations in the Field of Nanomaterials and Nanotechnologies, J. Heyrovsky Institute of Physical Chemistry, Academy of Sciences of the Czech Republic, Dolejskova 3, 182 23 Prague, Czech Republic; eliska.mikyskova@jh-inst.cas.cz

**Keywords:** polypropylene, crosslinking, aerogel, thermally induced phase separation, freeze-drying, supercritical drying

## Abstract

Polypropylene-based aerogels with high surface area have been developed for the first time. By chemical crosslinking of polypropylene with oligomeric capped-end amino compounds, followed by dissolution, thermally induced phase separation, and the supercritical CO_2_ drying process or freeze-drying method, the aerogels exhibit high specific surface areas up to 200 m^2^/g. Moreover, the silica-cage multi-amino compound was utilized in a similar vein for forming hybrid polypropylene aerogels. According to the SEM, the developed polypropylene-based aerogels exhibit highly porous morphology with micro-nanoscale structural features that can be controlled by processing conditions. Our simple and inexpensive synthetic strategy results in a low-cost, chemically resistant, and highly porous material that can be tailored according to end-use applications.

## 1. Introduction

Aerogels represent a class of extremely porous materials made by replacing the liquid portion of gels with air by a drying technology such as supercritical fluid drying (SCF) and freeze-drying. Aerogels have been attracting research attention due to their unique properties such as their high surface areas, extremely low densities, and low thermal conductivities [1]. Due to the high extent of porosity, aerogels have been shown to be promising candidates in a wide range of applications such as catalysis [2], chemical sensing [3], energy storage devices [4], and medical applications [5]. Generally, aerogels have been prepared from precursors having either organic or inorganic units in the backbone structure. Traditional silica aerogels invented by Kistler in the early 1930s [6] have been the most extensively studied but their utilization has several limitations such as sensitivity to moisture or fragility. Kistler also introduced the concept of SCF to prevent dimensional shrinkage and preserve the network morphology of wet gels into the final dry objects. Several works have been devoted to the modification of silica aerogel with an organic component with the prospect of expanding their potential application. This approach resolves the above-mentioned issues of silica aerogels by endowing the hydrophobicity and mechanical strength of the system [7,8,9]. Recently, novel soft silicone aerogels fabricated through cost-effective ambient temperature/pressure drying process were reported [10,11]. These aerogels exhibit wide-temperature mechanical flexibility and super-hydrophobicity display exceptional thermal insulating performance and show efficient oil absorption and separation capacity for various solvents and oil from water. Polymer aerogels emerged in 1989 [12] and offer improved mechanical properties, flexibility in chemical functionalities, and easy processing. Nowadays, these aerogels are used for applications that cannot be ensured by silica aerogels. Several types of polymer aerogels have been synthesized based on synthetic and natural polymers. Regarding polymer aerogels, polyimide- [13,14,15,16,17,18] or polyurethane-based [19,20,21,22] aerogels are the most notable, with potential utilization as a low dielectric substrate for antennas, which are desirable for small aircraft or urban air mobility vehicles. However, they have been prepared from costly precursors and often include complicated structures in the backbones.

Surprisingly, examples of the fabrication of porous materials based on low-cost, chemically resistant, and hydrophobic polypropylene (PP) are relatively scarce in the literature. For example, Erbil et al. [23] reported a superhydrophobic polypropylene gel-like porous coating with a water contact angle of 160°. Lin et al. [24] described macroporous membranes with bicontinuous structures including the phase diagram for the studied system. Polypropylene composite materials reported by Zhang et al. [25] and Li et al. [26] were shown to have excellent oil–water separation performance and lipophilic organic loading properties, respectively. However, in a later case, the specific surface area was measured to be only 38.66 m^2^ g^−1^. In a similar vein, Wang and Uyama [27] reported a macroporous PP sponge with utilization as an oil sorbent that has the potential to replace commercial nonwoven PP fabrics. Here, the authors claimed the excellent mechanical properties of the PP material; however, the material is not compressible in its dry state and the flexibility was induced only when it was immersed in organic liquid. Recently, Saleem et al. [28] reported the preparation of trimodal sponge from recycled polypropylene with effective sorption and sufficient tensile strength for practical applications. Park et al. [29] used a porous PP scaffold for the preparation of PP/silica composite aerogel with enhanced mechanical robustness and thermal insulation. Here, the specific surface area (based on BET) was reported to be only 57.2 m^2^ g^−1^ for composite material and 0.42 m^2^ g^−1^ for pure PP monolith. Similarly, Yoda et al. [30] integrated silica aerogel into PP foam producing a composite material with a thermal conductivity as low as silica aerogel monolith. Improved performance for the separation of reactive dye using a supported liquid membrane process was reported by Othman et al. [31] using a PP macroporous membrane, which was proved as an effective support for the separation of reactive Red 3BS from an aqueous solution.

Clearly, more extensive utilization of PP-based 3D porous materials would be possible as PP provides properties such as easy processing, low cost, and low density. Potential functionalization of the PP chain and subsequent crosslinking might result in a polymer network serving as a precursor for aerogel fabrication with enhanced structural stability. Moreover, it was shown that PP crosslinking results in improved mechanical [32,33]. However, functionalization of the non-reactive PP backbone may be challenging. Accordingly, maleic anhydride-grafted PP may represent a valuable springboard for constructing the polymer network. Herein, we present the synthesis of PP crosslinked polymers and their processing into aerogel materials with high surface areas up to 200 m^2^/g. To the best of our knowledge, this is the first report of PP-based aerogel material with meso/macroporous structure.

## 2. Experimental Section

### 2.1. Materials

iPP-*g*-MA—Isotactic polypropylenes grafted with 1 wt.% of maleic anhydride (**P1**, commercialized as G3003, Eastman^TM^*,* Kingsport, TN, USA), 3 wt.% of maleic anhydride (**P2**, commercialized as G3015, Eastman^TM^), 9 wt.% (**P3**, Merck), crosslinkers **C1**–**C3** (Merck, Darmstadt, Germany), and a crosslinker **C4** (Huntsman, Woodlands, TX, USA) commercialized as Jeffamines^®^ were used as received. Crosslinker **C5** was prepared by a known literature procedure [34]. Solvents for synthesis and workup obtained from commercial suppliers were used without any further purification.

### 2.2. Characterization

IR spectra were recorded on a FTIR spectrometer Nicolet iZ10 (Thermo Scientific, Waltham, MA, USA) with a DTGS detector using the ATR technique, spectral resolution of 4 cm^−1^, and spectral range of 4000–400 cm^−1^. Solid-state NMR measurements were recorded at 9.4 T using a JEOL JNM-ECZ400R/M1 (Tokyo, Japan) spectrometer. Samples were packed into 3.2-mm ZrO_2_ rotors for ^13^C (Larmor frequency of 100.52 MHz) measurements, with α-glycine (^13^C: 176.03 ppm; carbonyl signal) used as an external reference. Samples were recorded using ^13^C CP/MAS experimental conditions, acquired at a MAS of 20 kHz, 600 scans, optimized recycle delay of 6 s, and a cross-polarization (CP) contact time of 2 ms with high-power ^1^H decoupling for the removal of heteronuclear coupling. MestReNova software (v. 14.3.0) was subsequently used for the processing of the obtained spectra.

The porosity and total specific surface area were measured by the N_2_ adsorption method at the boiling temperature of liquid nitrogen (77 K). Prior to adsorption experiments, samples were evacuated at 40 °C for 12 h. Since the studied material can undergo decomposition at temperatures above 50 °C, this relatively low temperature of evacuation was chosen. The total surface area was evaluated using the BET (Brunauer–Emmett–Teller) method. The porosity distribution and average width of pores were evaluated by the BJH (Barrett–Joyner–Halenda) and DFT (Density Functional Theory) methods using the N_2_-DFT model with a regularization of 0.1. The experiments were performed with the 3Flex instrument (Micromeritics, Norcross, GA, USA) and the measured parameters were evaluated using Flex software (Micrometrics, Version 6.01). The structural analysis of samples was performed using UHR-FE-SEM Zeiss Ultra Plus. The samples were fixed on aluminum stubs using double-sided carbon tape; the samples were not modified or metal-coated before the analysis. Gentle beam conditions, including a low accelerating voltage of 0.5 kV and low probe currents > 1 pA were used to obtain images because of beam sensitivity and the low average atomic number of the observed material. The images in topographical contrast were acquired using a highly efficient InLens secondary electron detector.

### 2.3. Typical Procedure for the Synthesis of Crosslinked PP Materials

iPP-*g*-MA, **P1**–**P3** (5 g) was dissolved in *p*-xylene (100 mL) at 120 °C (30–60 min). Then, a crosslinking agent (**C1**–**C4**, equimolar ratio of NH_2_:maleic anhydride) dissolved in *p*-xylene (20 mL) was slowly added and the stirred resulting mixture was allowed to react at 120 °C for 3 h. After, the temperature was raised to 175 °C (oil bath) and the mixture was refluxed overnight. After cooling to r. t., acetone (50 mL) was added, the mixture was filtered on sintered glass, and the solid was rinsed with acetone (3 × 50 mL). The products were dried in vacuo in order to remove the acetone. This procedure gave the white–off-white powders in the isolated yields of 5–7 g. Notably, similar results were obtained when the tandem catalytic system (Et_3_N/acetic anhydride) was used at a lower temperature. The crosslinking reaction of **C5** and iPP-*g*-MA (**P2**) was performed following the above procedure. Only one extra step was added; the 50% **C5** solution was first diluted five times with propanol and then added dropwise to the PP-*g*-MA/*p*-xylene solution. Without dilution, the **C5** solution was too thick and thus impossible to disperse in the iPP-*g*-MA/xylene solution.

Assignment of **PXCY** refers to polymer/aerogel produced from polymer **PX** and crosslinker **CY**.

### 2.4. Typical Gelation Procedure

Powdered samples were dissolved in *p*-xylene (typically 100 mg/mL) at 120 °C (30–60 min). After, the systems were quenched to a desired temperature to solidify and poured into cellulose tubes saturated with xylene—this was airtight in plastic tubes. The gels, which formed within 30 min, were aged in the molds. The solvent within the gels was then gradually exchanged to acetone in 24-h intervals starting with 25% *p*-xylene in acetone, followed by pure acetone (3×).

### 2.5. Drying

**Figure 1:**
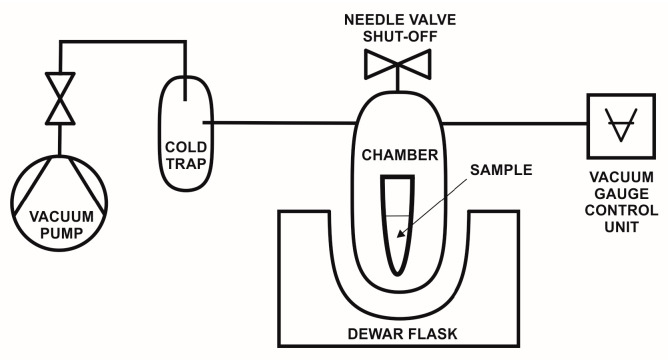
Diagram of the freeze-drying apparatus.

The samples were placed in chambers flushed with liquid carbon dioxide at laboratory temperature and pressure of 70 bar and kept overnight. After, the pressure was increased to 100 bar and the excess of the solvent was slowly released. After 2 h (solvent-carbon dioxide exchange), the flow of gaseous carbon dioxide was set to 1 L/min for 30 min. The cycles of 2 h of standing and 30 min of flushing were repeated three times and after the last standing cycle, the temperature was increased to 45 °C and the pressure was kept at 100 bar (to ensure scCO_2_). After temperature stabilization, the depressurization of the chamber was set to 0.5 bar/min, keeping the temperature above the critical point (45 °C). After several hours, the heating was turned off and the samples were removed from the reactor. In parallel, we used our home freeze-drying equipment (liquid nitrogen, 0.3 mbar) and obtained promising results that resemble supercritical drying. The device (Figure 1) consisted of a rotary oil pump, which was connected to the cold trap via a separation valve, which protected the pump against oil contamination. At the end of the system, there was a stainless steel vacuum chamber (75 mL) with an additional vacuum port enabling vacuum level measurement. Additionally, a needle valve was used to slowly aerate the system after the solvent extraction process. The samples were placed in a chamber filled with liquid nitrogen and then the entire chamber was immersed in liquid nitrogen to completely cool down. In the next step, the system was evacuated to a 0.3 mbar and kept overnight.

## 3. Results and Discussion

**Figure 2:**
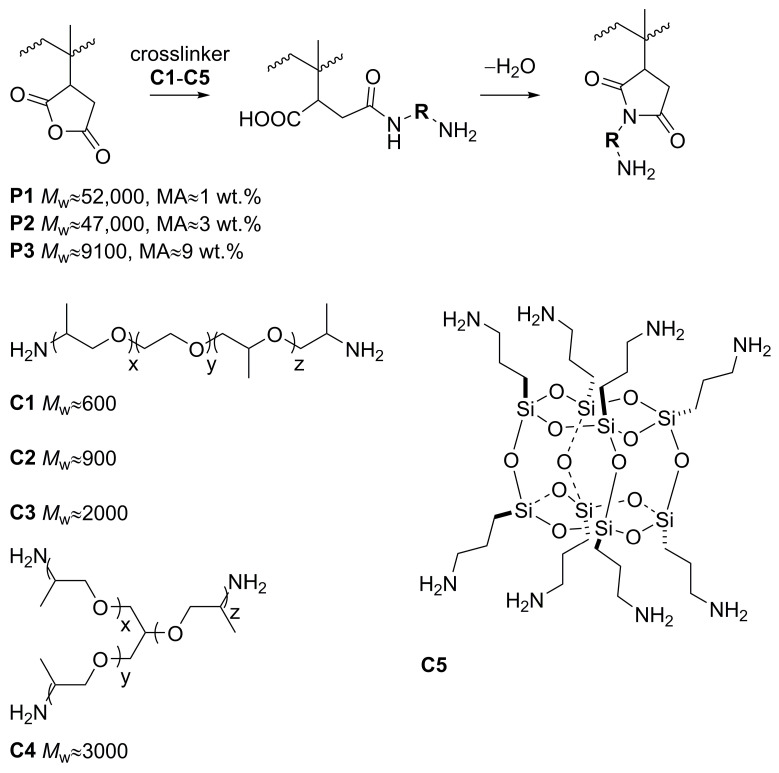
Reaction path leading to the crosslinked PP and structures of the crosslinkers.

**Figure 3:**
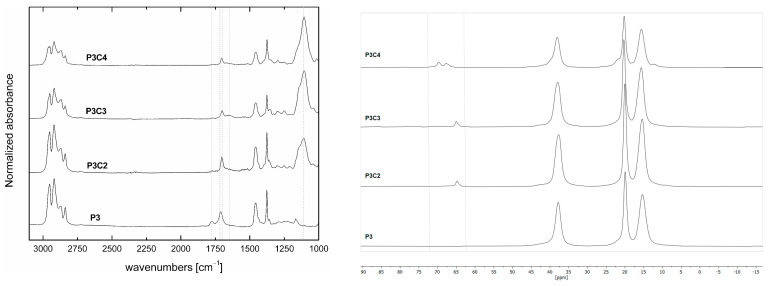
Examples of FTIR spectra (**left**) and ^13^C CP MAS spectra (**right**) of the crosslinked polymer **P3**.

Three different maleated isotactic polypropylenes **P1**–**P3** were utilized in crosslinking reactions, see Figure 2. The crosslinking agents, capped-end triblock polyether diamines, and triamines **C1**–**C4** were used in equimolar ratio with respect to their functional groups (NH_2_ and maleic anhydride, MA) and produced a 3D molecular network. Moreover, the bulk multi-amine compound **C5** was utilized in a similar vein and investigated separately. This kind of reaction has already been utilized using a barrel extruder [32,33]. However, we have optimized catalyst-free reaction conditions in xylene solvent, achieving the full reaction conversion of starting materials. The reaction between maleic and amino groups leads to an amide and carboxylic acid in the first step. In the second slower step, cyclization occurs and imide is formed, see Figure 2. The FTIR analyses were performed to check the chemical conversion of the materials. The example of recorded spectra is presented in Figure 3. Vibrational bands of parent polymer **P3** at 1774 and 1712 cm^−1^ can be assigned to the carbonyl vibrations of MA and carboxylic acid group, respectively, formed by hydrolysis. Upon the reaction with the amino group, the new carbonyl vibrations located at 1703 and 1655 cm^−1^ appeared, which can be assigned to imide and amide bonds, respectively. The amide bonds result from the opening of the anhydride ring/reaction with the carboxy group without cyclization. Vibrational bands located at 1107 cm^−1^ correspond with C–O vibrations of polyether chains. The successful integration of the crosslinker is further supported by ^13^C CP/MAS NMR (Figure 3). Besides the dominant signals of aliphatic carbons of the PP chain, the spectra of the crosslinked PP contained signals at around 65–70 ppm, ascribable to ether carbons of crosslinkers. These are well distinguishable in the case of polymer **P3C4**, showing peaks in approximately equal proportion at 69.7 and 67.7 ppm, which can be assigned to -O-**C**H_2_-CH_2_- and -O-**C**HCH_3_- ether carbons of the crosslinker **C4**.

The PP-based aerogels were prepared by the thermally induced phase separation (TIPS) method. In this method, homogeneous solutions were first prepared at elevated temperatures and after that, the phase separation occurred in a short time through cooling of the polymer solution. After, the diluent in wet gels was replaced by acetone unless otherwise noted. The resulting gels were then dried using either the scCO_2_ or freeze-drying method. Notably, we have found, during our study, that these methods provide comparable results. Freeze-drying is a facile one-step process without using any complicated techniques; thus, it is of technological significance.

**Figure 4:**
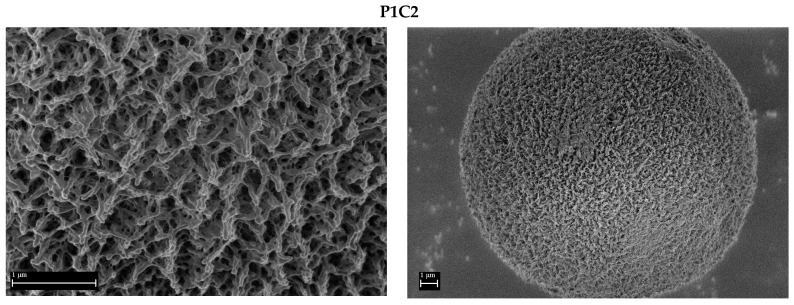

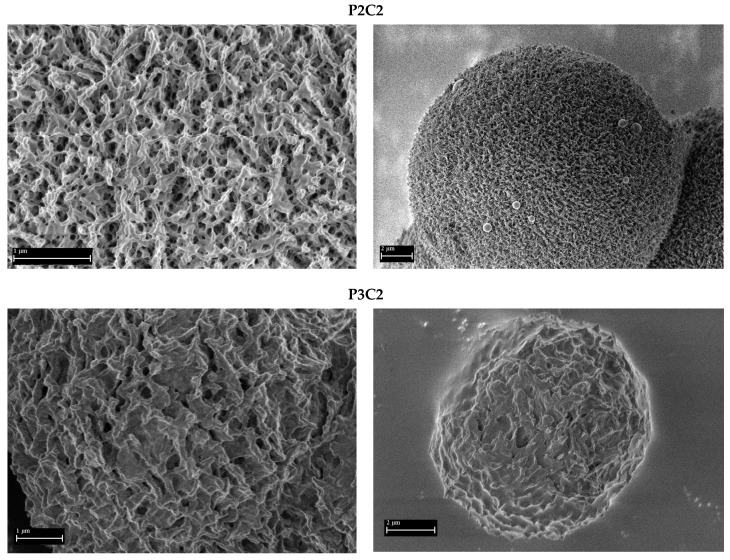
Example of SEM micrographs of aerogels based on crosslinked PPs.

The examples of SEM micrographs of the PP-based aerogels are shown in Figure 4. The highly porous nanostructural features of interconnected porous structures are evident in the higher magnifications (left). The agglomerated spherical microparticles formed by the precipitation of the PP polymers when the solution is cooled to room temperature are shown at lower magnification (right) and have a size of several micrometers. Based on the obtained results, all PP-based aerogels exhibit similar morphologies and pore shapes. However, polymer **P3**, with the highest density of maleic groups on the polymeric chain, combined with crosslinker **C2**, produced aerogel with the lowest degree of porosity (Figure 4, **P3C2**), which is most probably due to the denser packing of polymer chains. The morphological result is also correlated with BET results (*vide infra*), which show the lowest values of specific surface areas for polymer **P3**. 

Noticeable differences in morphologies and pore structures of aerogels are reflected in the literature comparison. For example, the PP membrane reported by Othman et al. [31] prepared by the TIPS method using diphenyl ether as a diluent showed cellular pore morphology with a pore size of around 9–17 μm. The authors also found that polymer concentration and quenching temperature considerably affect the final morphology of the membrane. Similarly, the PP sponge reported by Wang and Uyama [27] prepared by TIPS using a mixed solvent of decaline and 1-butanol had a macroporous structure with an average pore size of ca. 5 μm. This sponge was shown to be a promising material for the separation of oil from water because of its 3D interconnected macroporous structure and high hydrophobicity. By repeating the absorption process several times, the organic liquid was separated from water completely. The PP aerogel prepared by precipitation and lyophilization of isotatactic polypropylene solution (xylene) was reported by Li et al. [26] and the BET surface area was measured to be 38.66 m^2^ g^−1^. The material also showed a relatively large pore-size distribution. The pore diameter was calculated to be 22.5 nm, suggesting that the PP aerogel consists of mesopores. The morphology evaluated by SEM showed that the PP aerogel is composed of agglomerated micrometer-sized microgel particles. However, the authors did not report micrographs at higher magnifications so nanostructural features are unclear. Nevertheless, the material showed high lipophilic organic loading up to 1060 wt%. 

**Figure 5:**
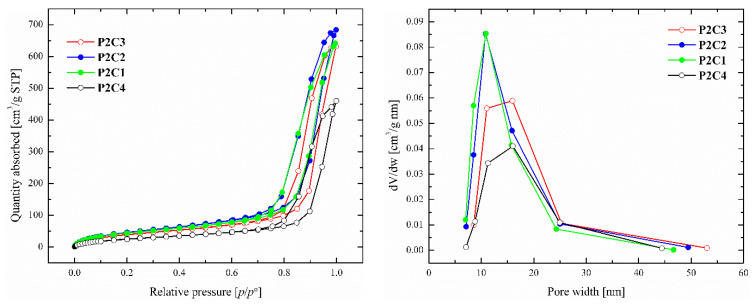
N_2_ adsorption–desorption isotherms of selected PP-based aerogels (**left**) and their pore-size distribution profiles (**right**).

The surface areas and pore characteristics of PP-based aerogels were evaluated by nitrogen absorption and desorption measurements. The N_2_ adsorption–desorption isotherms at 77 K and the pore-size distribution profiles are shown in Figure 5. The obtained data on surface area, pore volume, and average pore width are summarized in Table 1. 

The N_2_ absorption isotherms do not reach a well-defined saturation plateau at high relative pressures, suggesting some degree of macroporosity. The shapes of the narrow hysteresis loops are characterized by parallel branches, suggesting the presence of cylindrical pores with narrow pore-size distribution [35]. The pore volume and average pore width were determined using the desorption branches of isotherms by applying the Barrett–Joyner–Halenda (BJH) model [35].

In terms of the specific surface area size, it is possible to divide the studied samples into two groups. The surface area of the first group ranges from 100 to 220 m^2^/g (**P1C2**, **P2C1**, **P2C2**, **P2C3**, and **P2C4**). The shapes of the isotherms show that the materials are mesoporous with an average pore width between 11 and 16 nm. 

On the other hand, the samples of the second group (**P1C3**, **P1C4**, and **P3C3**) possessed low surface area and sample **P3C2** can be described as non-porous. Samples **P1C3** and **P1C4** have a comparable average pore width with the first group; however, the specific surface area and pore volume are significantly lower. This could be due to the different arrangement of structural units and formation of surface defects during the process of synthesis of materials, which corresponds to the observation obtained from SEM. Polymer **P3C4** possesses very poor solubility and was not utilized in aerogel preparation. Furthermore, from Table 1 it can be seen in terms of BET characteristics that **P1C2** aerogel obtained by supercritical drying provided similar values as the same aerogel obtained by the freeze-drying method. As freeze-drying is a facile one-step process without using any complicated techniques, it is in our interest for further preparation and investigation of aerogels.

When compared with the literature that reported on PP-based porous materials, we found that the aerogel materials reported here (*i*) show significantly higher specific surface areas, reaching up to over 200 m^2^/g and (*ii*) show very narrow pore-size distributions. In this regard and with respect to the literature reports on PP-based porous materials, it can supposed that the aerogels from crosslinked PP would even more effectively implement and load the organic lipophilic species inside to the PP aerogel network. 

**Figure 6:**
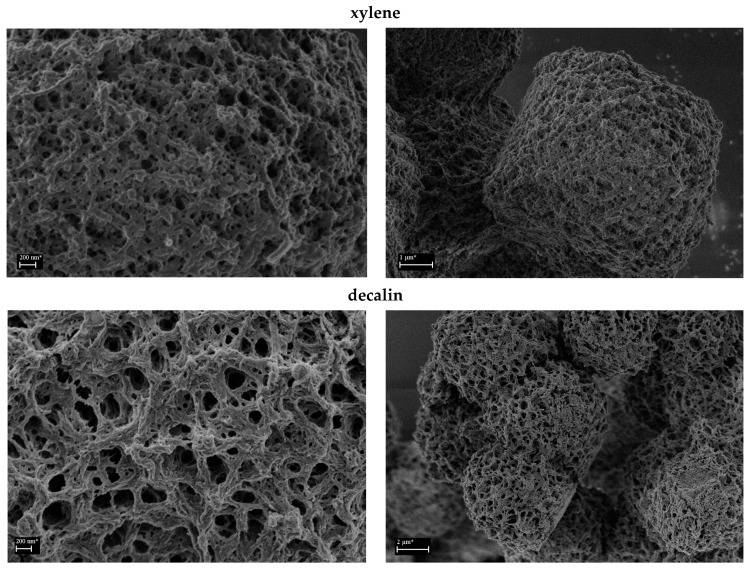

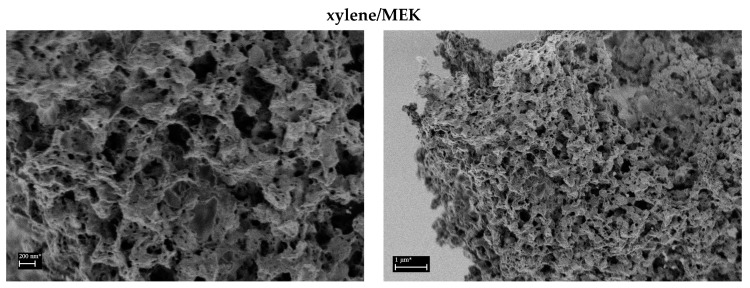
SEM micrographs of aerogels from **C5**-crosslinked polymer **P2** (**P2C5**) processed under different solvent conditions. * The magnification of the image corresponds to a Polaroid 545 print with the image size of 89 × 114 mm.

The examples of SEM micrographs of the **C5**-based aerogels of the polymer **P2** obtained by freeze-drying are shown in Figure 6. Here, the effects of the solvent, quenching temperature, molar ratio of crosslinker **C5**, and polymer concentration were studied. The solvent showed a large effect on the nanostructural features. The similar agglomerated microparticles were formed when xylene was used in the gel preparation (top). Also, similar spongy aggregates were obtained in the case of decalin (middle). However, in higher magnification, the structure resembles fibrillates more. The very different picture was provided when mixing the good solvent xylene with a nonsolvent methyl ethyl ketone (MEK), see Figure 6 bottom. The nonsolvent acting as a polymer precipitator decreases the crystallization time and thus provides much smaller aggregates. Also, the nonsolvent is responsible for increasing the nucleation rate and the crystal nuclei developed into smaller spherulites and fibrillates.

**Figure 7:**
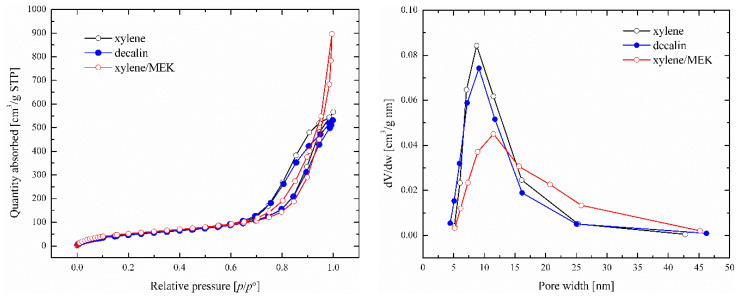
N_2_ adsorption–desorption isotherms of **P2C5** aerogels prepared under different solvent conditions (**left**) and their pore-size distribution profiles (**right**).

The BET results for the **P2C5** aerogels are summarized in Table 2. Examples of N_2_ absorption–desorption isotherms and pore-size distribution profiles are shown in Figure 7. It can be seen that very similar values of the specific surface area, pore volume, and average pore width were achieved even under different aerogels preparation conditions. The specific surface area ranged from 185 to 206 m^2^/g. The average pore width possessed relatively uniform values and, together with the shape of the N_2_ isotherm, indicated the mesoporous structure of the prepared aerogels. Only the aerogel prepared using a non-solvent MEK differed very significantly in terms of surface and porosity properties. The addition of MEK caused the formation of much smaller aggregates compared to the other samples, resulting in significant changes in porosity.

## 4. Conclusions

In summary, we reported a group of highly porous polypropylene-based materials with a specific surface area of up to 200 m^2^/g, which is significantly higher with respect to the previous research made into the polypropylene porous materials. This is the first time that PP-based porous materials have been produced using freeze drying as a facile one step process, which can be easily scaled-up and provides results comparable with the supercritical drying process. It was also shown that micro-nanostructural features of PP-based aerogels can be controlled by processing conditions. This study provides a low-cost solution for the preparation of highly porous materials that might offer versatility in its functionality and may open a new possibility for further exploration and design of high-performance materials for environmental remediation purposes as an example.

## Figures and Tables

**Table 1:** Summary of specific surface area, pore volume, and average pore width of studied samples obtained by supercritical drying. ^a^ Obtained by freeze-drying for comparison.

Polymer	Crosslinker	Specific Surface Area [m^2^/g]	Pore Volume [cm^3^/g]	Average Pore Width [nm]
**P1**	**C2**	216/163 ^a^	1.17/0.71 ^a^	13/11 ^a^
**P1**	**C3**	38	0.30	14
**P1**	**C4**	10	0.06	15
**P2**	**C1**	173	0.99	11
**P2**	**C2**	185	1.04	14
**P2**	**C3**	154	0.97	16
**P2**	**C4**	102	0.68	16
**P3**	**C2**	<1	0.0004	6
**P3**	**C3**	13	0.01	3

**Table 2:** Summary of the specific surface area, pore volume, and average pore width of **P2C5** aerogels prepared under different conditions and obtained by freeze-drying. ^a^ Molar ratio of MA:NH_2_. Higher molar ratio than 50% leads to poorly soluble material. ^b^ Using 50 mg/mL instead of 100 mg/mL. ^c^ Xylene/MEK 4/6 (*v*/*v*) is the respective gel that is directly dried (without acetone extraction). ^d^ Dried at r. t.

Molar Ratio of C5 [%] ^a^	Solvent	Quenching Temperature [°C]	Specific Surface Area [m^2^/g]	Pore Volume [cm^3^/g]	Average Pore Width [nm]
30	xylene	25	192	0.76	11
50	xylene	25	198/203 ^b^	0.84/0.88 ^b^	11/11 ^b^
50	xylene	4	193	0.78	11
50	xylene	80	200	0.87	11
50	decalin	25	185	0.80	12
50	xylene/MEK ^c^	25	206/67 ^d^	1.39/0.44 ^d^	21/12 ^d^

## Data Availability

The original contributions presented in the study are included in the article/Supplementary Materials, further inquiries can be directed to the corresponding author.

## Supplementary Materials

The following supporting information can be downloaded at: https://www.mdpi.com/article/10.3390/polym16101382/s1, Figures S1–S3: FTIR spectra, Figures S4-S10: N2 absorption-desorption isotherms, Figures S11–S21: SEM micrographs.
