# Supplementary material for: Preparation and Investigation of High Surface Area Aerogels from Crosslinked Polypropylenes
Source: Polymers (Basel). 2024 May 12;16(10):1382. doi: 10.3390/polym16101382 (PMC11125074; doi:10.3390/polym16101382)
Supplement: Supplementary file 1 [file polymers-16-01382-s001.zip › polymers-2957307-supplementary.pdf]

Preparation and Investigation of Aerogels from Crosslinked Polypropylenes

Radek Coufal,[a,b*] Mateusz Fijalkowski,[b] Kinga Adach,[b] Huaitian Bu,[c] Christian W. Karl,[c] Eliška Mikysková[d] and Stanislav Petrík[b]

[a]*Department of Science and Research, Faculty of Health Studies, Technical University of Liberec, 461 17 Liberec, Czech Republic*

[b]*Department of Advanced Materials, Institute for Nanomaterials, Advanced Technologies and Innovation (CXI), Technical University of Liberec, 461 17 Liberec, Czech Republic*

[c]*Department of Materials and Nanotechnology, SINTEF Industry, Forskningsveien 1, 0373 Oslo, Norway*

[d]*Center for Innovations in the Field of Nanomaterials and Nanotechnologies, J. Heyrovsky Institute of Physical Chemistry, Academy of Sciences of the Czech Republic, Dolejskova 3, Prague 18223, Czech Republic*

radek.coufal@tul.cz

Supporting Information

TABLE OF CONTENTS

IR spectra 2

BET results 3

SEM micrographs 6

IR spectra

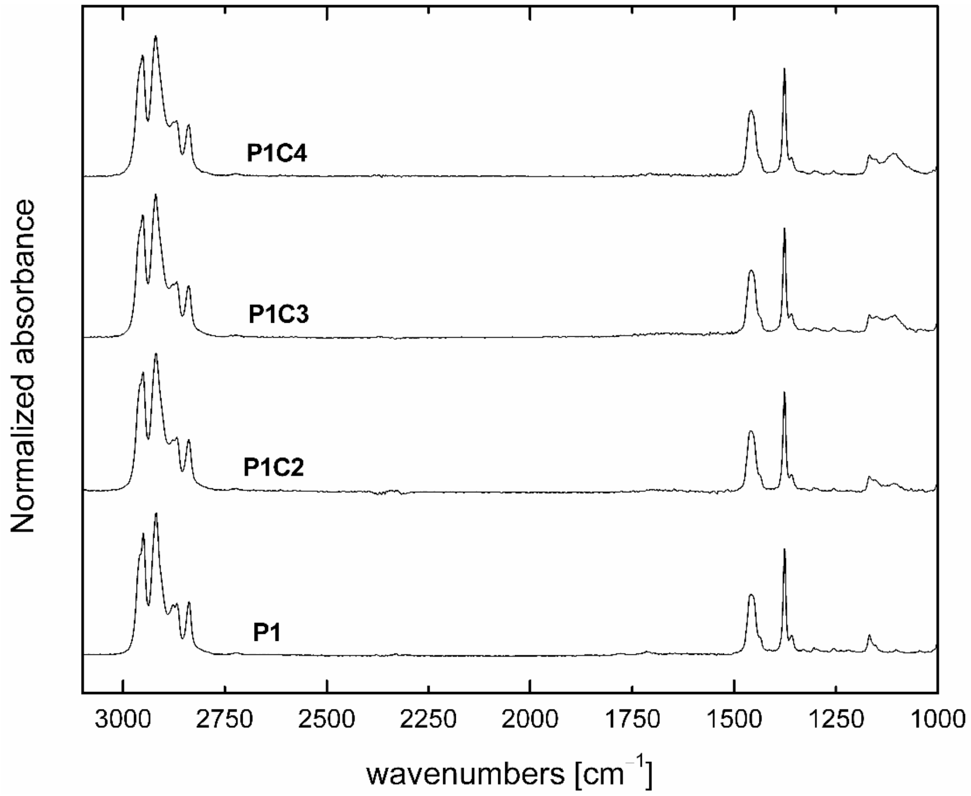

Figure S1 FTIR spectra of the crosslinked polymer **P1**.

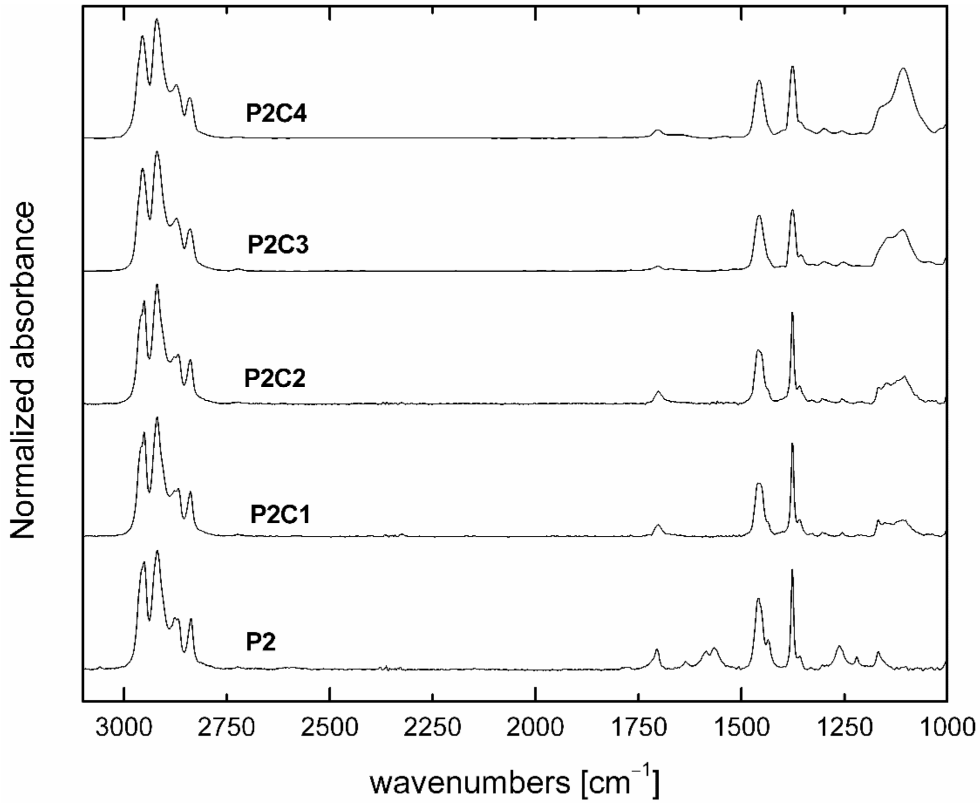

Figure S2 FTIR spectra of the crosslinked polymer **P2**.

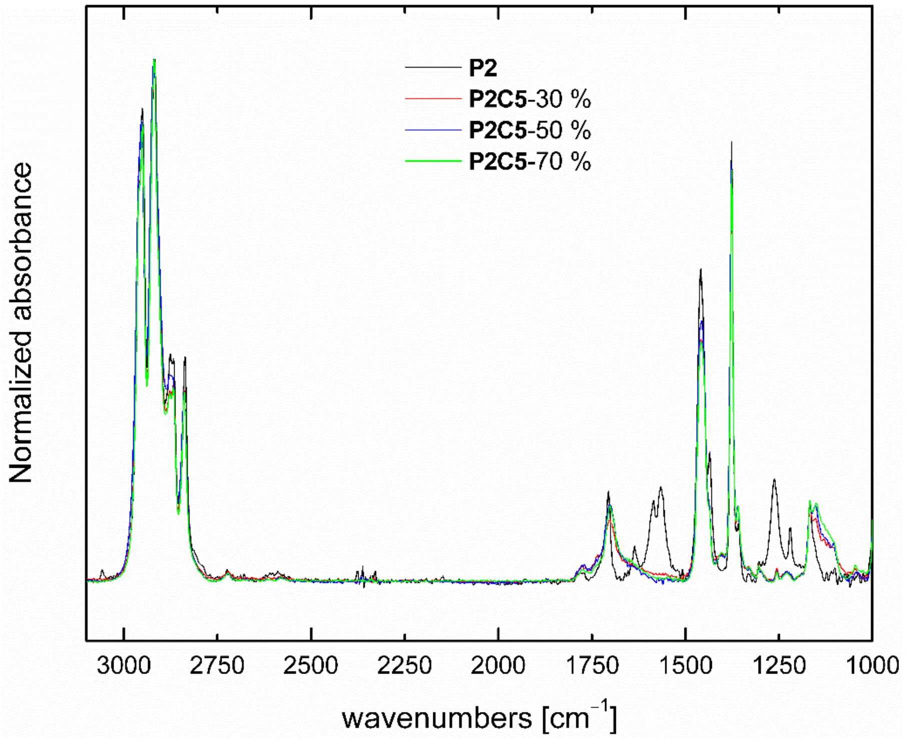

Figure S3 FTIR spectra of the crosslinked polymer **P2** with the crosslinker **C5** in different molar ratio of MA:NH_2.

BET results

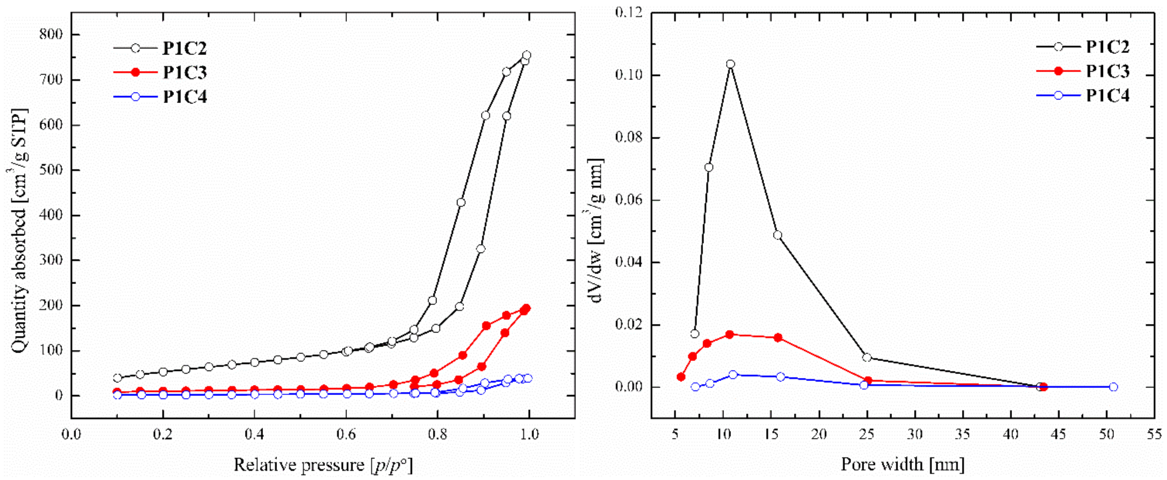

Figure S4 N_2 adsorption-desorption isotherms of aerogels from the polymer **P1** (left) and their pore-size distribution profiles (right).

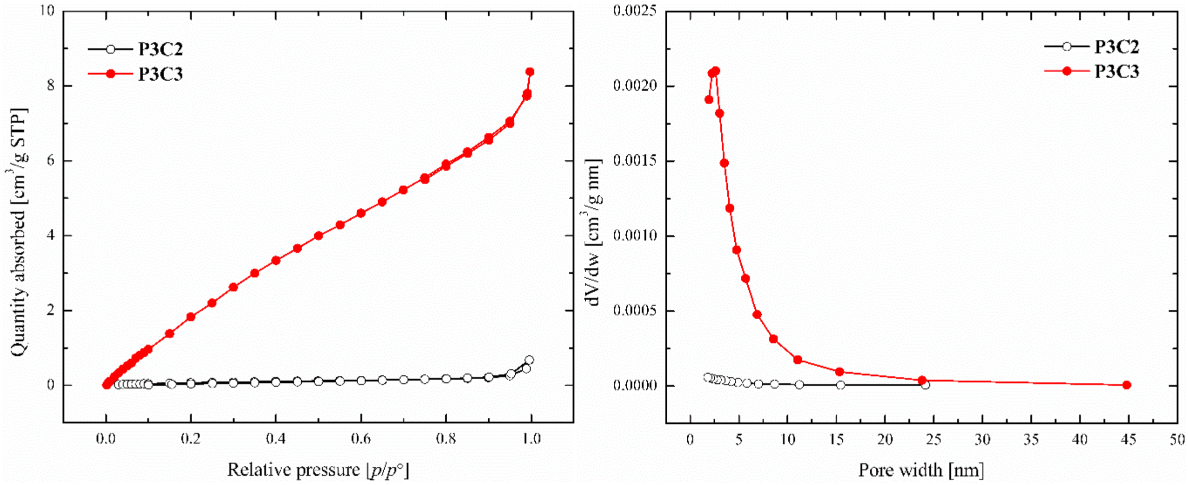

Figure S5 N_2 adsorption-desorption isotherms of aerogels from the polymer **P3** (left) and their pore-size distribution profiles (right).

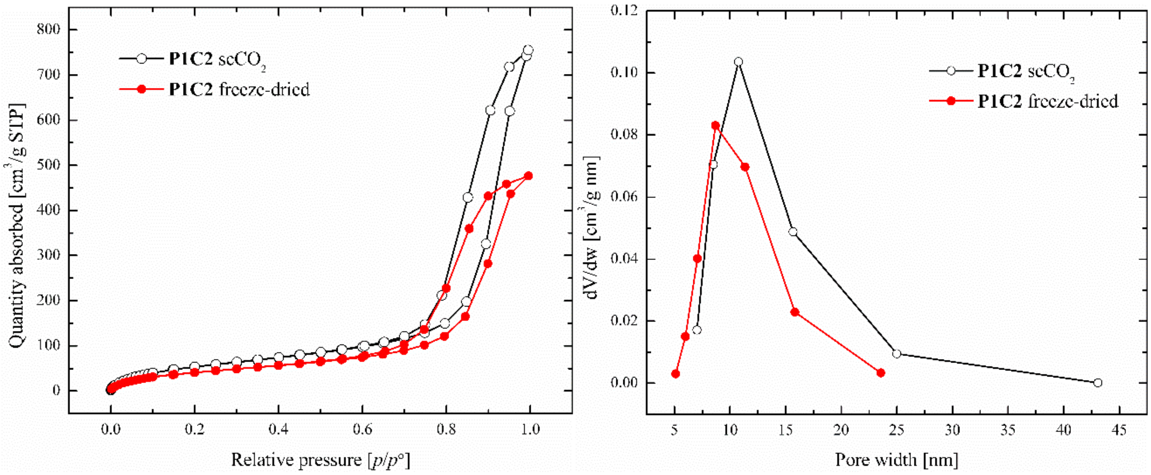

Figure S6 N_2 adsorption-desorption isotherms of **P1C2** aerogels obtained by $scCO_2$ and freeze-drying (left) and their pore-size distribution profiles (right).

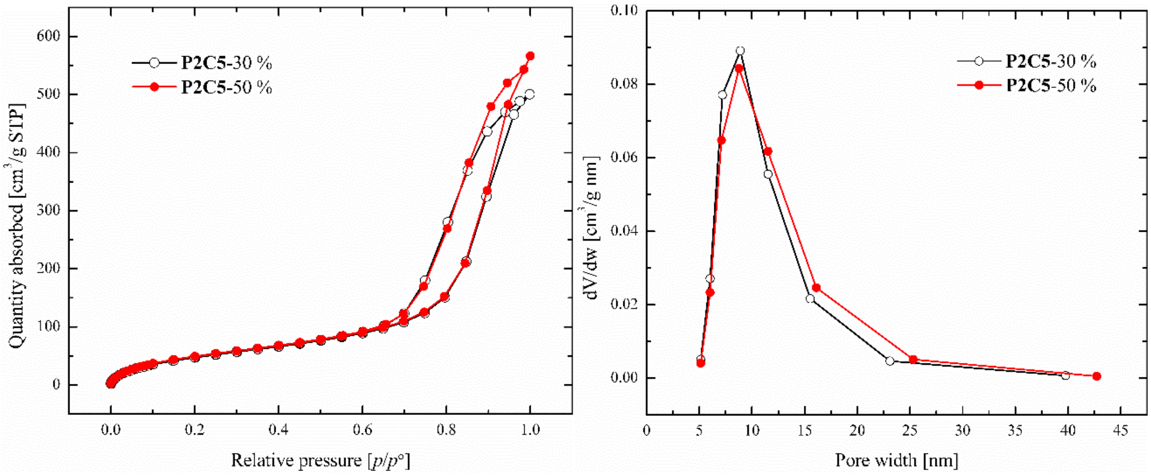

Figure S7 N_2 adsorption-desorption isotherms of **P2C5** aerogels using different molar ratio of $MA:NH_2$ in polymer synthesis (left) and their pore-size distribution profiles (right).

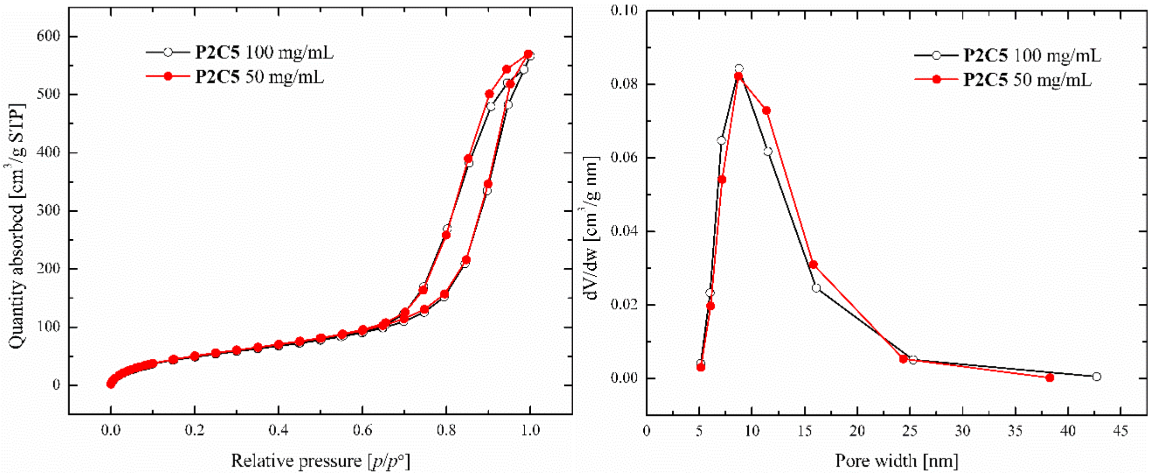

Figure S8 N_2 adsorption-desorption isotherms of **P2C5** aerogels (50% molar ratio of MA:NH_2) using different polymer concentration in gel preparations (left) and their pore-size distribution profiles (right).

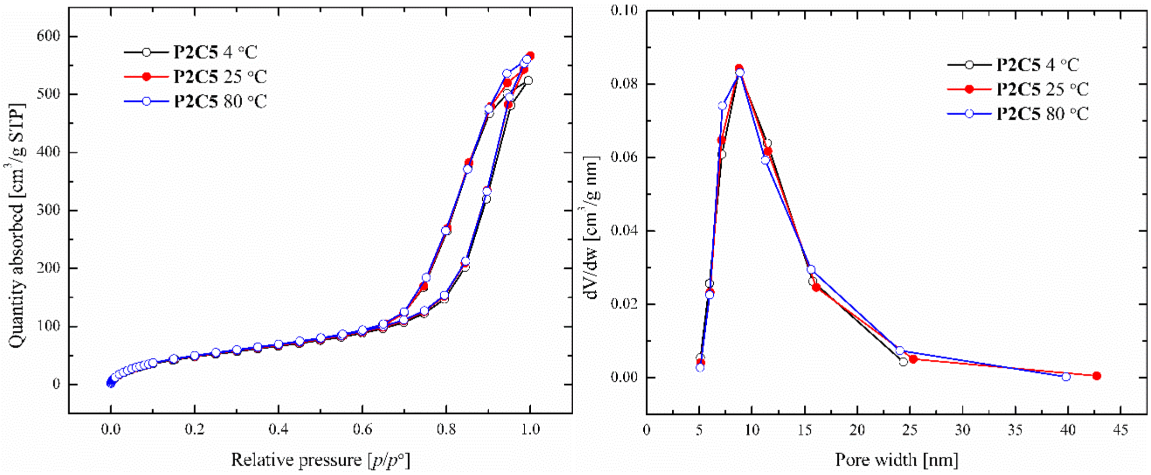

Figure S9 N_2 adsorption-desorption isotherms of **P2C5** aerogels obtained by using different quenching temperature in gel preparations (left) and their pore-size distribution profiles (right).

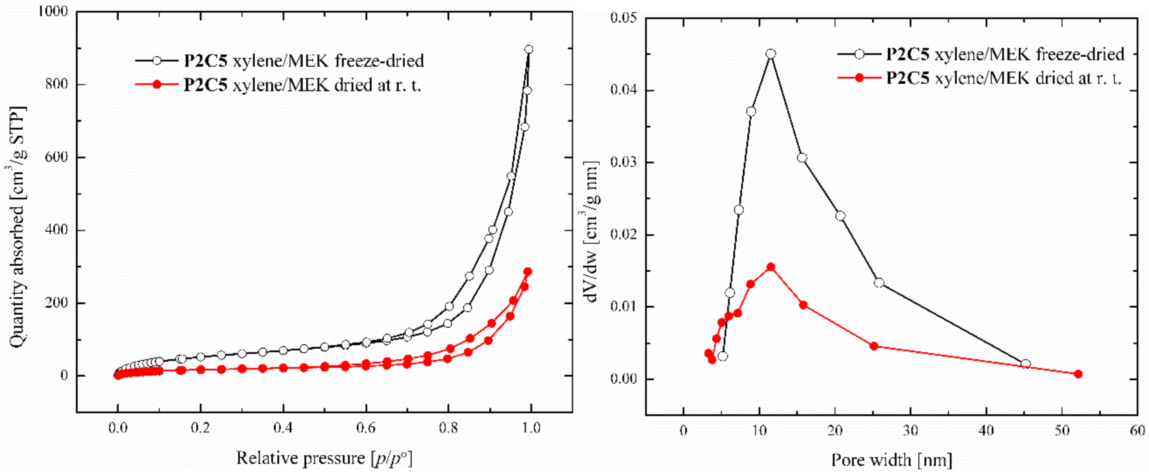

Figure S10 N_2 adsorption-desorption isotherms of **P2C5** aerogels obtained by freeze-drying and drying at r. t. of xylene/MEK gels (left) and their pore-size distribution profiles (right).

SEM micrographs

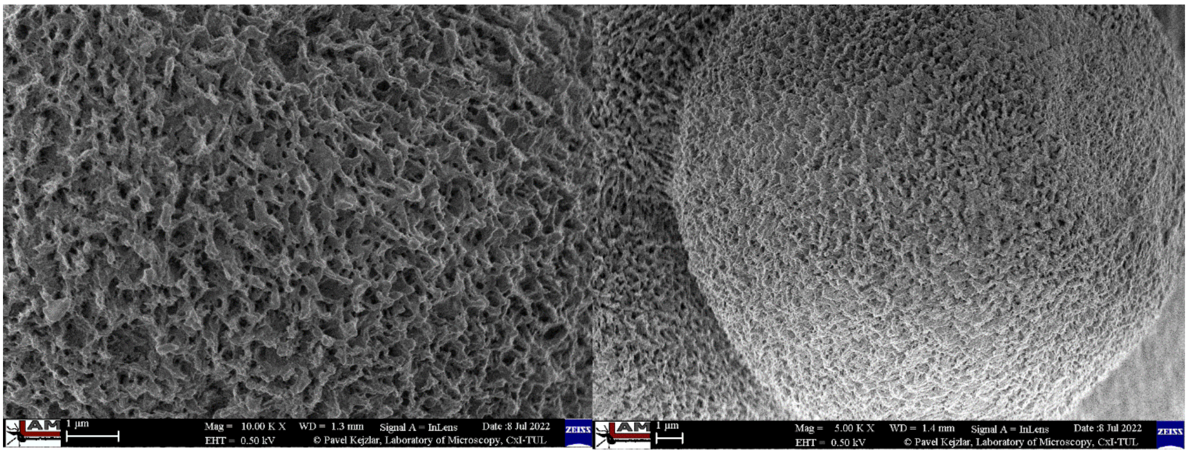

Figure S11 SEM micrographs of **P1C3** aerogel.

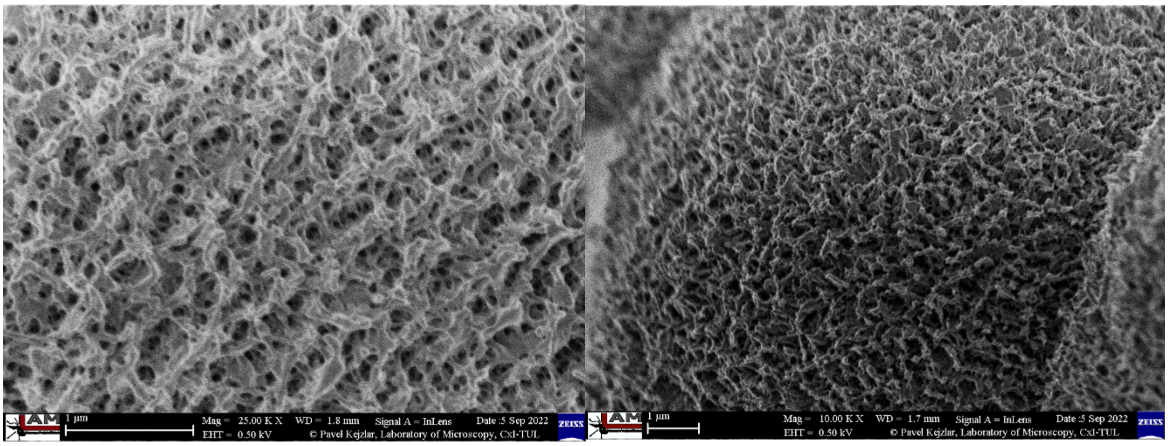

Figure S12 SEM micrographs of **P1C4** aerogel.

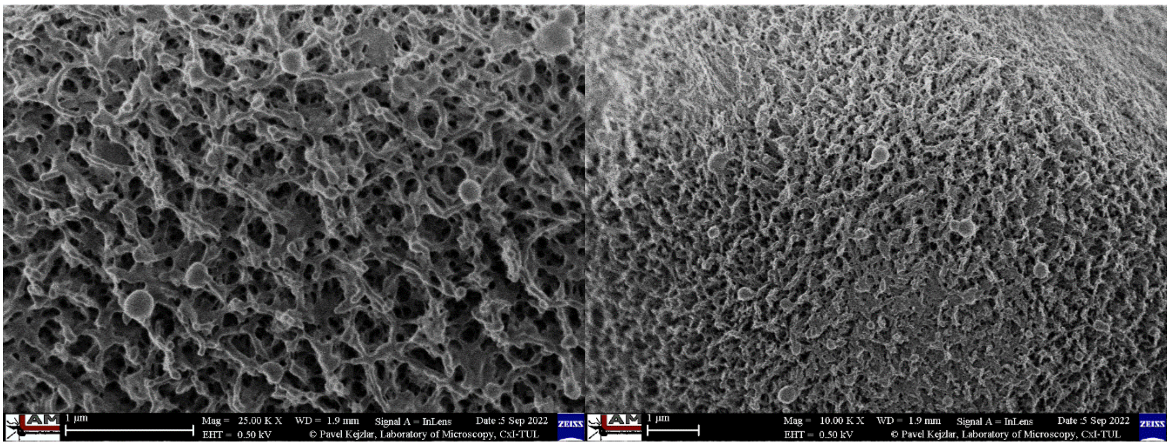

Figure S13 SEM micrographs of **P2C1** aerogel.

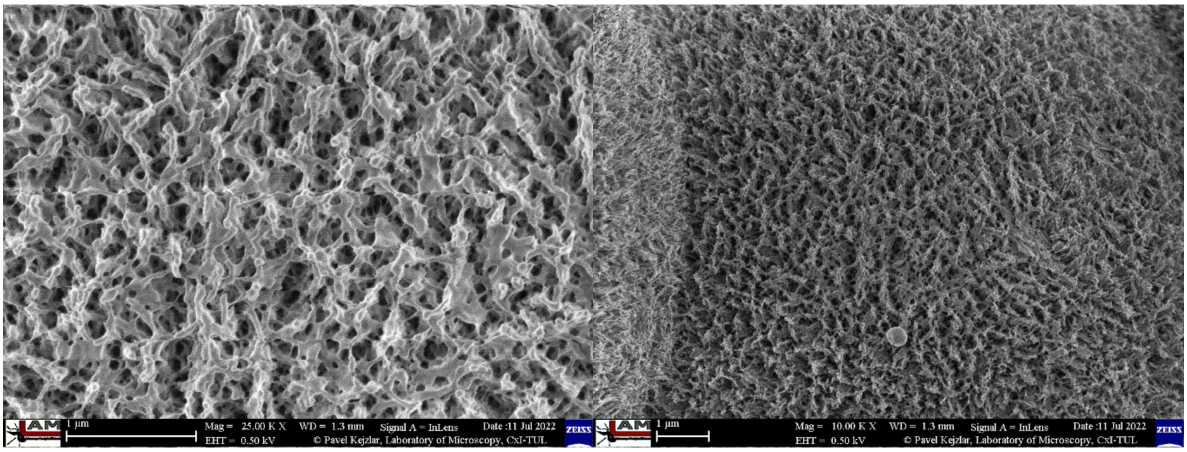

Figure S14 SEM micrographs of **P2C3** aerogel.

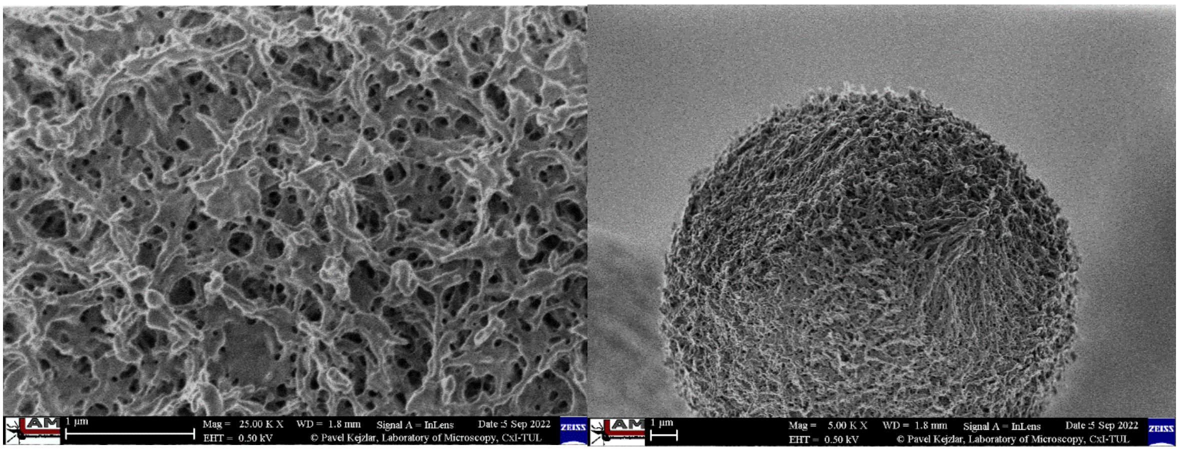

Figure S15 SEM micrographs of **P2C4** aerogel.

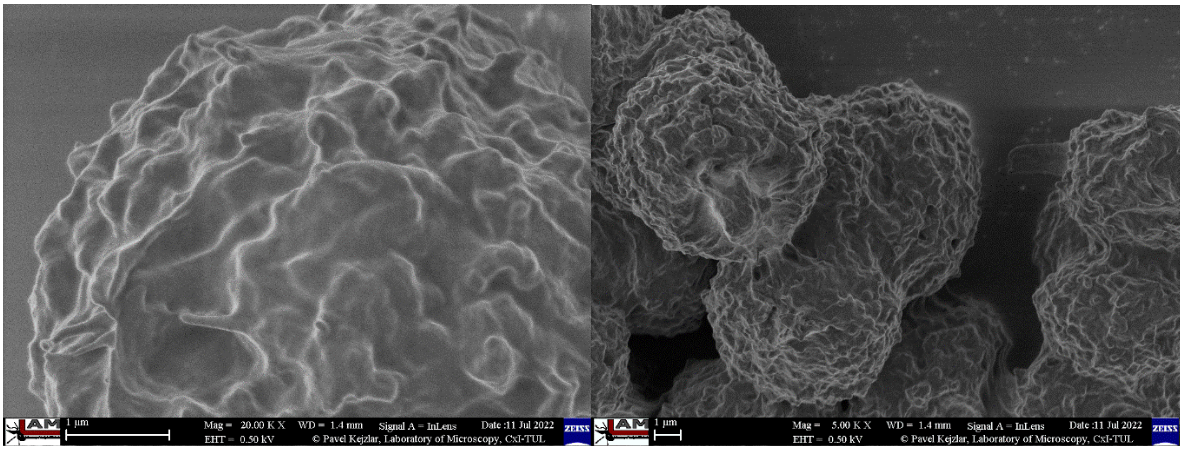

Figure S16 SEM micrographs of **P3C3** aerogel.

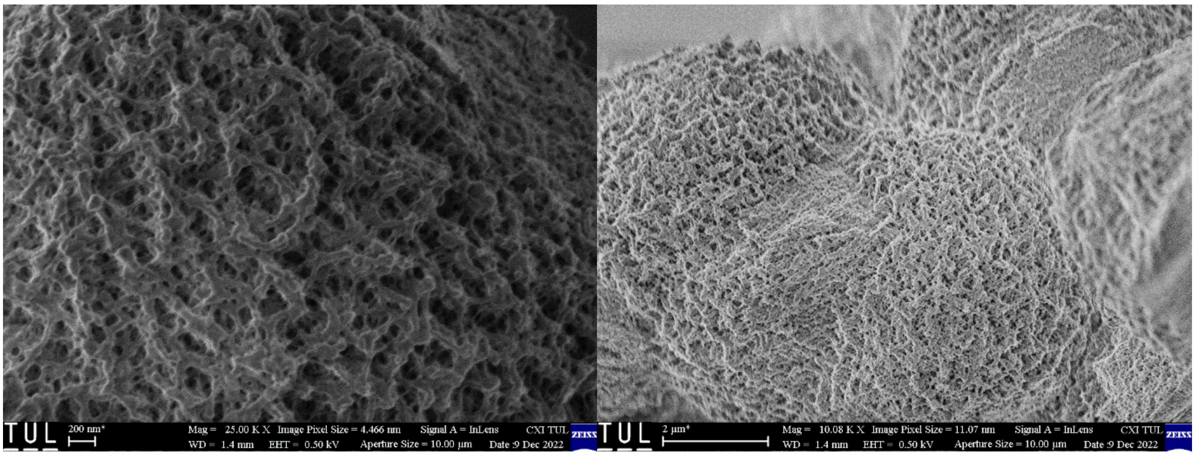

Figure S17 SEM micrographs of **P2C5** aerogel, 30% molar ratio of MA:NH_2, quenching temperature 25 °C.

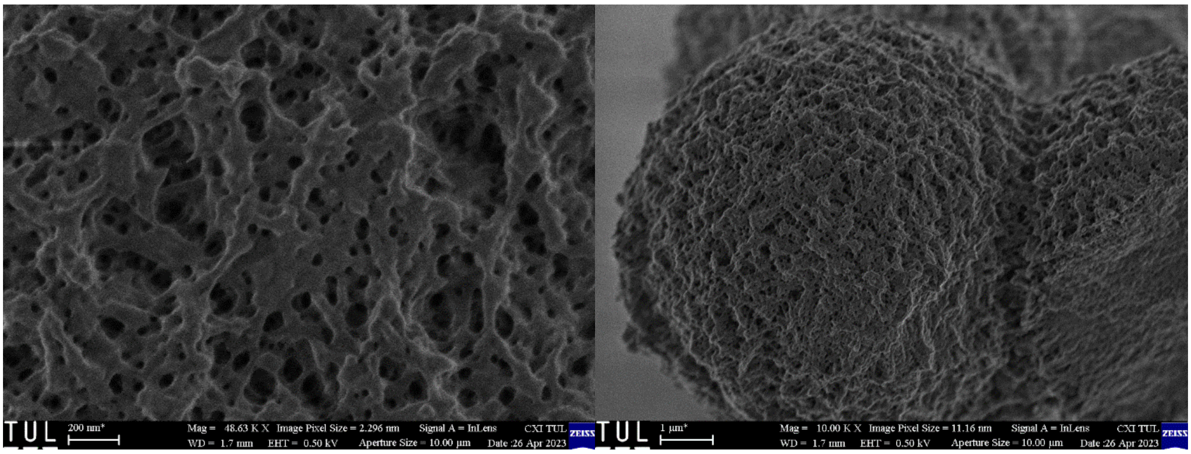

Figure S18 SEM micrographs of **P2C5** aerogel, 50% molar ration of MA:NH_2, quenching temperature 4 °C.

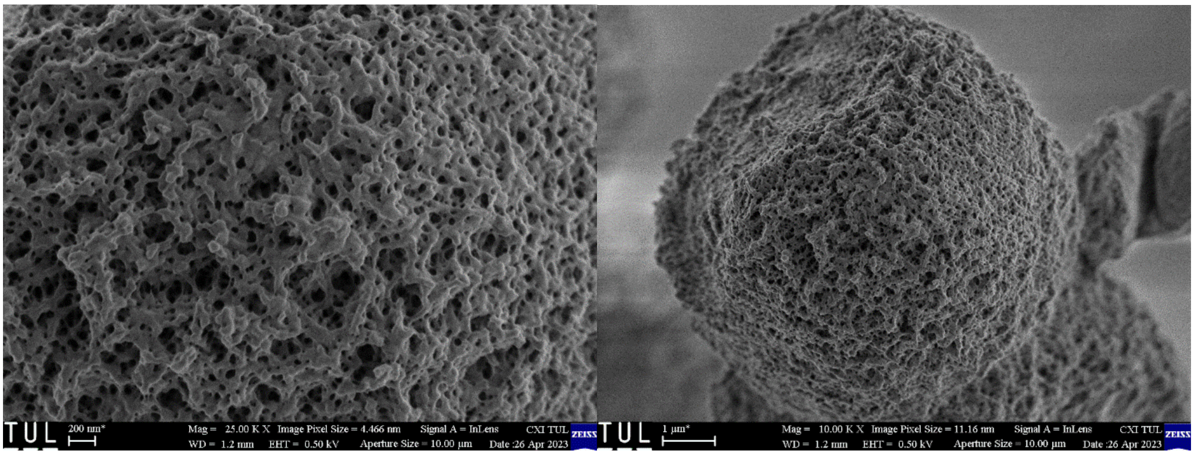

Figure S19 SEM micrographs of **P2C5** aerogel, 50% molar ration of MA:NH_2, quenching temperature 80 °C.

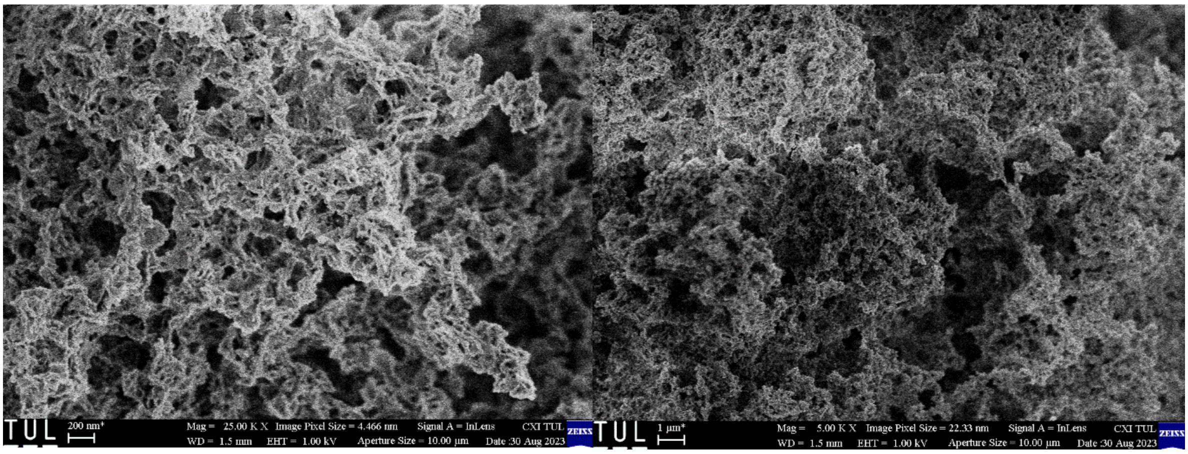

Figure S20 SEM micrographs of **P2C5** aerogel, 50% molar ration of MA:NH_2, using xylene/MEK (4/6, v/v) in gel preparation, dried at r. t.

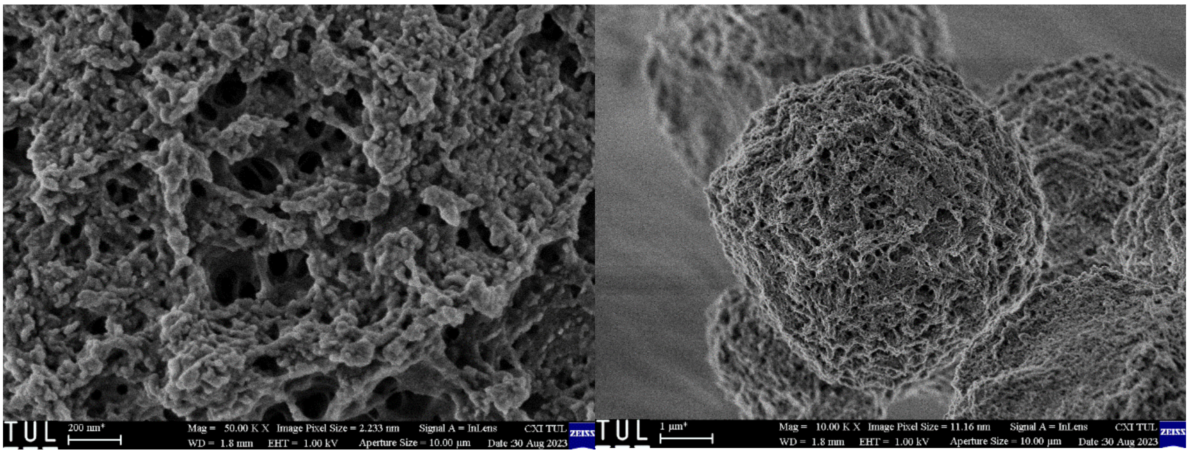

Figure S21 SEM micrographs of **P2C5** aerogel, 50% molar ration of MA:NH_2, using concentration of 50 mg/mL in gel preparation.
